# Comparison of Herpes Simplex Virus 1 Strains Circulating in Finland Demonstrates the Uncoupling of Whole-Genome Relatedness and Phenotypic Outcomes of Viral Infection

**DOI:** 10.1128/JVI.01824-18

**Published:** 2019-04-03

**Authors:** Christopher D. Bowen, Henrik Paavilainen, Daniel W. Renner, Jussi Palomäki, Jenni Lehtinen, Tytti Vuorinen, Peter Norberg, Veijo Hukkanen, Moriah L. Szpara

**Affiliations:** a Department of Biochemistry and Molecular Biology, Center for Infectious Disease Dynamics, and the Huck Institutes of the Life Sciences, Pennsylvania State University, University Park, Pennsylvania, USA; b Institute of Biomedicine, University of Turku, Turku, Finland; c Clinical Microbiology, Turku University Hospital and Institute of Biomedicine, University of Turku, Turku, Finland; d Department of Virology, University of Gothenburg, Gothenburg, Sweden; Northwestern University

**Keywords:** herpes simplex virus (HSV), comparative genomics, genetic variation, genotype, phenotype, phylogeny

## Abstract

A majority of adults in Finland are seropositive carriers of herpes simplex viruses (HSV). Infection occurs at epithelial or mucosal surfaces, after which virions enter innervating nerve endings, eventually establishing lifelong infection in neurons of the sensory or autonomic nervous system. Recent data have highlighted the genetic diversity of HSV-1 strains and demonstrated apparent geographic patterns in strain similarity. Though multiple HSV-1 genomes have been sequenced from Europe to date, there is a lack of sequenced genomes from the Nordic countries. Finland’s history includes at least two major waves of human migration, suggesting the potential for diverse viruses to persist in the population. Here, we used HSV-1 clinical isolates from Finland to test the relationship between viral phylogeny, genetic variation, and phenotypic characteristics. We found that Finnish HSV-1 isolates separated into two distinct phylogenetic groups, potentially reflecting historical waves of human (and viral) migration into Finland. Each HSV-1 isolate harbored a distinct set of phenotypes in cell culture, including differences in the amount of virus production, extracellular virus release, and cell-type-specific fitness. Importantly, the phylogenetic clusters were not predictive of any detectable pattern in phenotypic differences, demonstrating that whole-genome relatedness is not a proxy for overall viral phenotype. Instead, we highlight specific gene-level differences that may contribute to observed phenotypic differences, and we note that strains from different phylogenetic groups can contain the same genetic variations.

**IMPORTANCE** Herpes simplex viruses (HSV) infect a majority of adults. Recent data have highlighted the genetic diversity of HSV-1 strains and demonstrated apparent genomic relatedness between strains from the same geographic regions. We used HSV-1 clinical isolates from Finland to test the relationship between viral genomic and geographic relationships, differences in specific genes, and characteristics of viral infection. We found that viral isolates from Finland separated into two distinct groups of genomic and geographic relatedness, potentially reflecting historical patterns of human and viral migration into Finland. These Finnish HSV-1 isolates had distinct infection characteristics in multiple cell types tested, which were specific to each isolate and did not group according to genomic and geographic relatedness. This demonstrates that HSV-1 strain differences in specific characteristics of infection are set by a combination of host cell type and specific viral gene-level differences.

## Abstract (precis)

Herpes simplex viruses (HSV) infect a majority of adults. Recent data have highlighted the genetic diversity of HSV-1 strains and demonstrated apparent genomic relatedness between strains from the same geographic regions. We used HSV-1 clinical isolates from Finland to test the relationship between viral genomic and geographic relationships, differences in specific genes, and characteristics of viral infection. We found that viral isolates from Finland separated into two distinct groups of genomic and geographic relatedness, potentially reflecting historical patterns of human and viral migration into Finland. These Finnish HSV-1 isolates had distinct infection characteristics in multiple cell types tested, which were specific to each isolate and did not group according to genomic and geographic relatedness. This demonstrates that HSV-1 strain differences in specific characteristics of infection are set by a combination of host cell type and specific viral gene-level differences.

## INTRODUCTION

Human herpes simplex virus 1 (HSV-1; family *Herpesviridae,* genus *Simplexvirus,* species *Human herpesvirus 1*) has been recognized as a cause of major human disease since the era of Hippocrates (1), and to this day there is no effective vaccine (2). Approximately 50% of adults in Finland are seropositive for HSV-1 (3–5), and 13% are seropositive for HSV-2 (3, 6). HSV infects via epithelial or mucosal surfaces, after which it is taken up by nerve endings and establishes a lifelong infection in neurons of the sensory or sympathetic nervous system. From these neuronal sites the virus can reactivate, transit via nerve endings back to the skin surface, and reinitiate skin or mucosal shedding at the same site as the original infection. In addition to recurrent epithelial lesions, HSV also causes infectious keratitis, worsens acquisition and shedding rates for human immunodeficiency virus (HIV), and can progress to cause rare but life-threatening encephalitis (1).

Recent comparative genomics studies of HSV-1, from our lab and other labs, have demonstrated that HSV-1 strains from unrelated individuals can differ in 2 to 4% of the viral genome (7–13). HSV-1 has a large DNA genome of ∼152 kb, encoding >75 proteins (14, 15). Prior comparisons of HSV-1 strains based on single-gene or whole-genome sequencing have found at least three major clades that cluster geographically, with evidence of recombination between these groups (9, 16–18). However, these studies have focused almost exclusively on the quantification of sequence diversity, without connecting these observations to experimental measures of viral fitness and/or virulence. Several recent examples have demonstrated the usefulness of comparing closely related virus strains to explore the effects of minor sequence differences on phenotypic outcomes (10, 19–22). To date, publications examining the genome-wide diversity of HSV-1 in diverse clinical isolates have not connected these observations to any classic measures of viral fitness, such as viral replication in cells, or antiviral drug resistance (12, 13, 23). Based on the decades of prior research leading to all current antiviral approaches for HSV-1, we anticipate that a connection between viral genetic data, cell-based measures of viral fitness, and *in vivo* measures of viral pathogenesis will be crucial to drive the next generation of viral therapeutics. Examining the phenotypic differences displayed by HSV-1 strains in culture provides an opportunity to explore the scope of these differences and test whether or not they are linked to previously observed patterns of geographic diversity.

Finland has a unique history that has led to a relatively homogeneous and stable population (24–26), providing a unique view on the evolution of viruses that are persistent in the population. There has been gene flow contributing to the human population of Finland, both from the east and from the south (southwest) (24–26). The eastern migrations from Siberia began over 3,500 years ago, and allele sharing with modern East Asian populations can be observed even in present day Finns (25, 26). Finland also has one of the best genealogical databases in the world, which, in combination with computerized medical records and a high rate of patient participation, has led to many recent advances in human medical genetics (27, 28). This may enable future studies to explore the coevolution of human and viral genetic variation in this population.

We have characterized 10 HSV-1 strains isolated from a random subset of Finnish clinic visits and compared their growth properties, drug resistance, and other phenotypic features. Full-genome sequences of these viruses were compared to each other and to other previously described strains of HSV-1 to reveal patterns of interhost and intrahost variability. While two phylogenetic subgroups were detected at a genomic level, these subgroups were not predictive of any detectable pattern in the observed phenotypic differences, demonstrating that whole-genome relatedness is not a proxy for viral phenotype. We anticipate that these data will aid in efforts to develop improved sequence-based antiviral therapies by providing additional data on conserved versus divergent areas of the HSV-1 genome and contribute to development of a vaccine against HSV infections based on attenuation of these or related clinical isolates (29). These data present an opportunity to explore the diversity of chronic herpesviruses in the Finnish population and to lay the foundation for future studies that explore the connections between viral genetic differences, host genetic predispositions, and their potential relationship(s) to clinical outcomes of HSV-1 infection.

(This article was submitted to an online preprint archive [30]).

## RESULTS

### Comparison of growth properties of Finnish HSV-1 strains *in vitro*.

**FIG 1:**
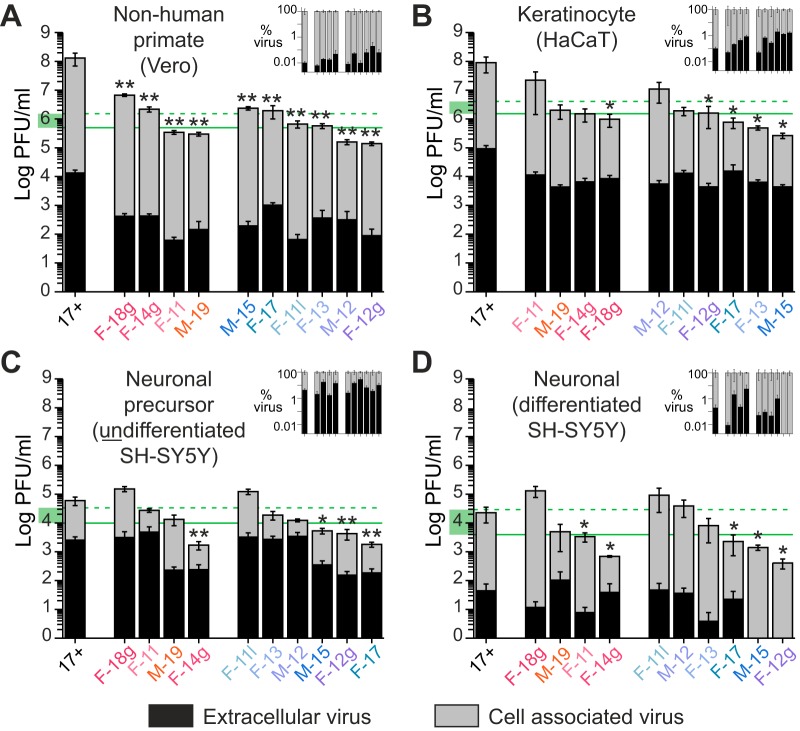
Comparison of growth properties of Finnish HSV-1 strains reveals the uncoupling of phenotypic and geographic variation. Histograms comparing virus production for 10 Finnish strains of HSV-1, compared to the HSV-1 reference strain 17+, in different cell types, as indicated. Vero monkey kidney cells are defective in interferon signaling. For ease of comparison, the strains are color coded and divided into the genomic subgroups used in later figures (illustrated in Fig. 3). Viruses are plotted in descending order based on their viral production levels within each cell type and subgroup. Each histogram bar plots infectious virions in the cell-associated fraction versus those released from cells (extracellular). Horizontal lines indicate the median (solid green) and the average (dashed green) amounts of virus production for the 10 Finnish HSV-1 strains in each cell type. The green box on the *y* axis highlights the multi-log decrease in viral production in panels C and D versus that in panels A and B. The inset in each graph shows a plot of extracellular released virus as a percentage of total virus production. Viruses in each inset are plotted in the same order as the matched panel graph. Bar shows standard errors of the means. *, *P* ≤ 0.05; **, *P* < 0.001, for comparison of results for the total virus amount (cell-associated plus extracellular) for each clinical isolate to that produced by the HSV-1 (17+) reference strain.

A random set of 10 circulating Finnish HSV-1 isolates was selected from residual laboratory diagnostic specimens (Table 1). We first examined *in vitro* phenotypic characteristics of these HSV-1 isolates by comparing their overall titers and rates of intracellular virus versus extracellular (released) virus production at 24 h postinfection (hpi) in a range of cell types (Fig. 1). These included nonhuman primate kidney-derived epithelial (Vero) cells (Fig. 1A), human keratinocyte (HaCaT) cells (Fig. 1B), and human neuroblastoma (SH-SY5Y) cells in a mixed, undifferentiated state (Fig. 1C) as well as in a differentiated, neuronal state (Fig. 1D). Compared to production levels in epithelial and keratinocyte cells, overall viral production was markedly reduced in neuronal precursor cells and differentiated neuronal cells (compare Fig. 1A and B to C and D). The wild-type HSV-1 reference strain 17+ replicated to significantly higher titers than any of the circulating clinical isolates in Vero cells, which are routinely used for HSV propagation, and, to a lesser extent, strain 17+ replicated at a higher titer in keratinocytes as well (Fig. 1) (*P* < 0.05; 10- to 100-fold-higher viral amounts). Different clinical isolates excelled at producing virions in each cell type, with no clear patterns of most- or least-efficient viral production or release across all cell types. The amount of extracellular released virus was less than 10% of the total virion production in all cell types except the undifferentiated neuronal precursor cells (Fig. 1, insets). Each virus strain was also tested for acyclovir (ACV) resistance. Despite some variation in ACV susceptibility (50% inhibitory concentration [IC_50_] values), none of the circulating Finnish viruses was considered resistant to ACV (Table 1). There were also no significant differences among the 10 clinical isolates in the plaque morphologies or the types of cytopathic effect induced in Vero cell cultures (data not shown).

**TABLE 1:** Finnish HSV-1 strains used for genome sequence comparisons

Sample no.	Gender of donor	Strain code	Age of donor (yr)	Lesion type or diagnosis	Acyclovir sensitivity (IC_50_ [μg/ml])^*a*^
H1211	Female	F-11	21	Blister	0.25
H1215	Male	M-15	5	Blister	0.03
H12113	Female	F-13	29	Blister	0.11
H12114	Female	F-14g	23	Genital blister	0.50
H12117	Female	F-17	57	Blister	0.15
H12118	Female	F-18g	19	Genital blister	0.13
H1311	Female	F-11*l*	59	Blister (lip)	0.31
H1312	Male	M-12	39	Blister	0.13
H1412	Female	F-12g	28	Genital lesion	0.09
H15119	Male	M-19	36	Blister	0.15

a The IC_50_ value was 8.08 μg/ml for the HSV-1 Δ305 strain (a thymidine kinase-negative, ACV-resistant control) and 0.13 μg/ml for the reference strain HSV-1 (17+). The limit of resistance was ≥2.0 μg/ml.

### Genetic and genomic analysis of Finnish HSV-1 strains.

**FIG 2:**
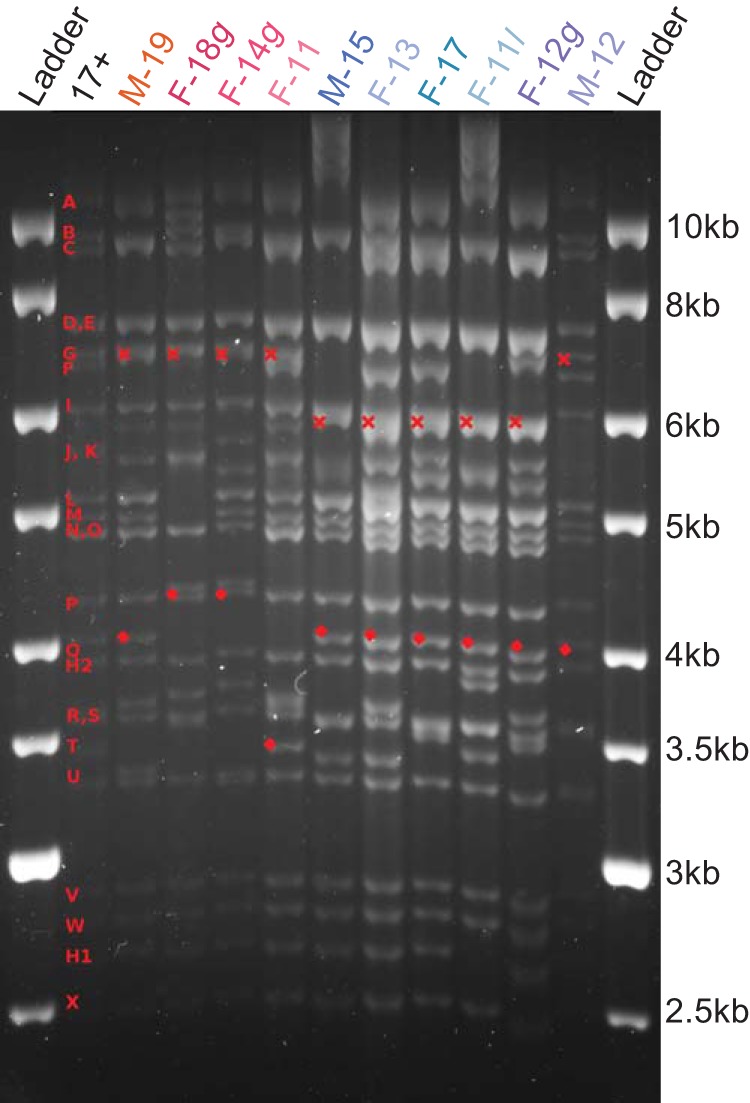
RFLP comparison reveals at least two subgroups of Finnish HSV-1 strains. Viral genomic DNA was digested with SalI and separated via electrophoresis to distinguish overall genetic patterns. Classically defined SalI fragment notation (red letters) is indicated on the left (58). Red crosses (×) indicate variation in the sizes of SalI fragment G, while diamonds indicate variations in the SalI Q fragment. For ease of comparison, the strains are color coded according to the same scheme as in later figures (illustrated in Fig. 3).

Based on prior data suggesting the effects of successive waves of migration on the human population in Finland (31, 32), we next assessed the overall genetic diversity of these 10 HSV-1 isolates. We first performed a restriction fragment length polymorphism (RFLP) analysis on viral genomic DNA, which revealed at least two broad patterns of variation (Fig. 2). The diversity of bands led us to examine these genetic differences with greater precision using high-throughput, deep genome sequencing (HTS) and comparative genomics analysis (see Materials and Methods for details). A previously described bioinformatics pipeline was used to *de novo* assemble a full-length consensus genome for each strain (Table 2). A viral consensus genome represents the most common nucleotide detected at each nucleotide position in that viral population. All viral genomes had an average coverage depth between 1,000× and 2,800×, with >96% of the genome covered at a depth exceeding 100× (Table 2). As observed in prior publications, the few areas with <100× coverage correlated with regions of high G+C content and/or highly repetitive sequences (9, 10, 33). An average coverage depth of >1,000× enabled us to compare the full genetic complement of the viral genome for each strain, to analyze differences both between and within each viral strain population, and to compare them to previously described viral genomes.

**TABLE 2:** Sequencing statistics for Finnish HSV-1 strains

Sample no.	Strain code	% viral genome >100×	Avg coverage	No. of raw sequence reads	No. of reads used for assembly	Input DNA purity: % viral DNA	GenBank accession no.
H1211	F-11	96	1,027×	4.2 million	2.8 million	66	MH999843
H1215	M-15	99	2,808×	5.5 million	3.8 million	70	MH999846
H12113	F-13	98	2,354×	3.8 million	2.4 million	64	MH999842
H12114	F-14g	98	2,078×	5.9 million	3.8 million	66	MH999844
H12117	F-17	98	1,976×	4.1 million	2.7 million	66	MH999845
H12118	F-18g	98	1,678×	4.2 million	2.9 million	69	MH999847
H1311	F-11*l*	99	2,541×	4.6 million	3.0 million	65	MH999848
H1312	M-12	98	2,415×	6.0 million	3.7 million	61	MH999849
H1412	F-12g	98	1,524×	4.5 million	3.1 million	69	MH999851
H15119	M-19	99	1,536×	4.8 million	3.0 million	64	MH999850

### Genetic relatedness of Finnish HSV-1 samples.

**FIG 3:**
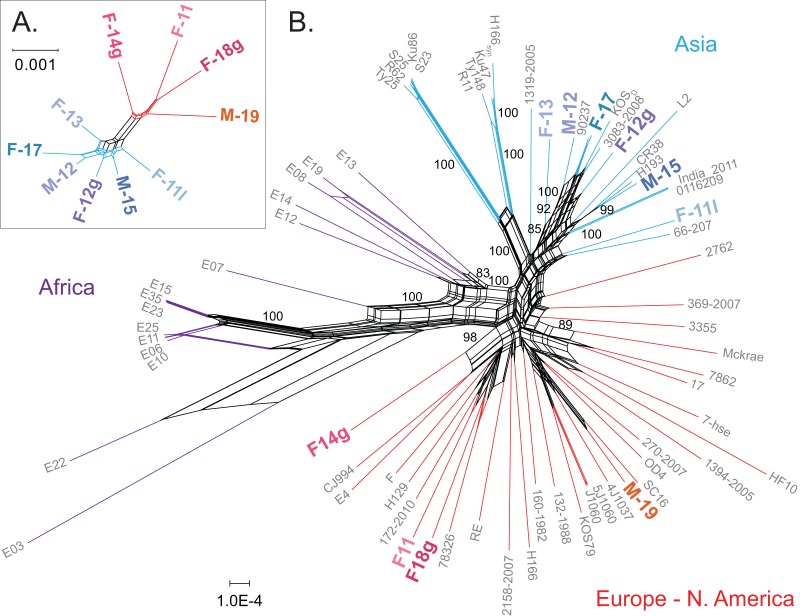
A phylogenetic network of genetic relatedness reveals that Finnish HSV-1 isolates separate into two subgroups. A phylogenetic network of genetic relatedness was constructed using SplitsTree4 to demonstrate how the 10 new Finnish HSV-1 isolates relate to each other (A) and to previously described HSV-1 genomes from a diversity of locations (B). Geographic clustering of branches and clades resembles previously described patterns (9, 16–18). Strains are color coded by origin: red for Europe and North America, blue for Asia, and purple for Africa. Finnish HSV-1 genomes are indicated in bold and separate into two subgroups, which appear to relate previously recognized Asian and European and North American clades. Bootstrap values larger than 80 supporting clades with three or more strains are shown on the network. The extent of recombination among HSV-1 strains is demonstrated by the parallel edges in the network and a phi test for recombination. The isolate name, country of origin, GenBank accession number, and reference(s) for each previously described HSV-1 genome are given in Table S1 in the supplemental material.

First, we used the viral consensus genomes to compare how overall variation in these 10 Finnish HSV-1 genomes related to the patterns observed by RFLP analysis (Fig. 2). Whole-genome alignments were created using the 10 Finnish HSV-1 genomes, as well as these 10 in conjunction with a diverse set of previously published HSV-1 genomes (see Materials and Methods; see also Table S1 for a full list [10, 34–38]). We then used SplitsTree to create a phylogenetic network that revealed the relatedness of the 10 Finnish viral genomes to each other (Fig. 3A) and to previously described HSV-1 genomes (Fig. 3B). This approach revealed that the Finnish HSV-1 genomes separated into two subgroups, which appear to relate to previously recognized Asian and European/North American clades. These data echo those of the initial RFLP analysis.

### Protein-coding variation in Finnish HSV-1 samples.

**FIG 4:**
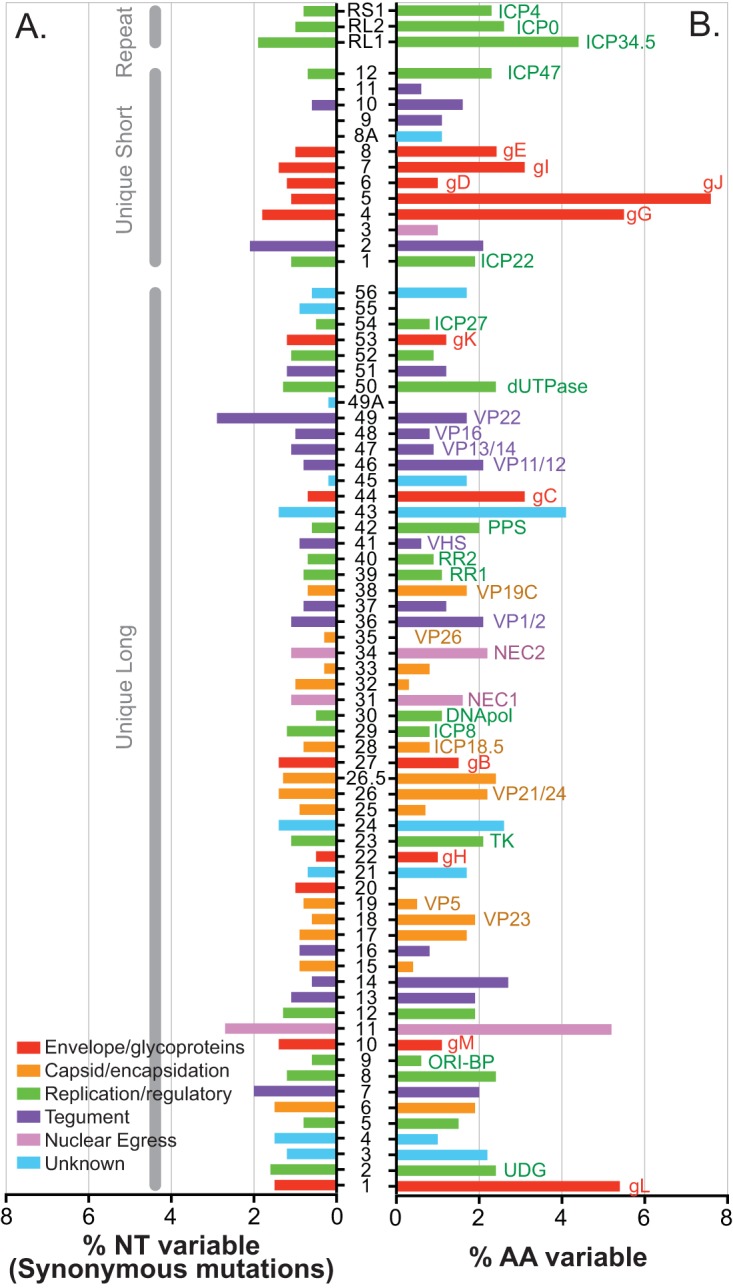
Substantial protein-coding variation exists between Finnish HSV-1 isolates. Histogram depicts the percentage of synonymous nucleotide (NT) differences per gene (A) and the percent amino acid (AA) variation per protein (B) for the 10 Finnish HSV-1 strains compared here. Labels on the left in panel A indicate the repeat, unique short, and unique long regions that contain each of the sequentially numbered viral coding regions. Where possible, the common name of each protein is shown to the right of the appropriate histogram bar in panel B, e.g., UL1 is also known as gL (bottom of graph). Nucleotide and amino acid differences were quantified at the consensus level of each viral genome. See Tables S2 and S3 in the supplemental material for additional data on the number of nucleotide differences and the ratio of nonsynonymous to synonymous evolutionary differences detected in each gene and in the virus strains used in the calculations.

Next, we examined these HSV-1 isolates for more fine-scale genetic differences which are invisible at the level of RFLP analysis. Here, we examined DNA and amino acid alignments for each protein-coding region of the HSV-1 genome (Tables S2 and S3). We found that from 0 to 8% of each coding region differed at the amino acid level between the 10 Finnish viral genomes (Fig. 4). Only a few small coding regions (UL20, VP26 [UL35], UL49A, and UL55) showed no amino acid variation between viral isolates. Consistent with previous findings (9), gL (UL1), UL11, UL43, gG (US4), and gJ (US5) were among the most divergent proteins. Together, these interstrain genetic differences in 70 HSV-1 proteins, shown in Fig. 4, provide ample opportunities to generate the phenotypic diversity observed in the data of Fig. 1. Overall, these levels of amino acid coding diversity reflect those seen in previous analyses (9) and between other known HSV-1 genomes.

### Minor allelic variants in Finnish HSV-1 viral populations.

**FIG 5:**
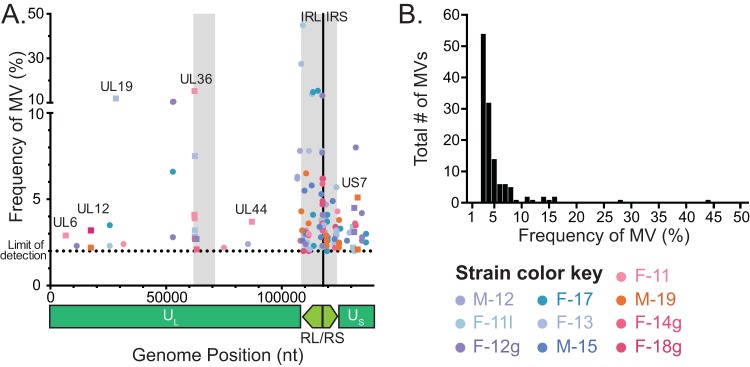
Scatter plot illustrates the location and low frequency of minor variants in each Finnish HSV-1 genome. (A) Scatter plot demonstrates the position and frequency of minor variants detected in each Finnish HSV-1 genome. Spheres on the graph indicate minor variants in intergenic regions. Squares with gene names indicate MVs located in genes. Strains are indicated according to the color key at the lower right. A genome diagram is shown below the *x* axis for reference. Areas containing large numbers of tandem repeats, e.g., around UL36 in the unique long (U_L_) region and throughout the repeat short/repeat long (RS/RL) region, are denoted as gray blocks on the graph. The limit of detection was set at 2% (see Materials and Methods for details). (B) Histogram depicts the total number of minor variants in each frequency bin, summed across all 10 Finnish HSV-1 strains. Bins are in 1% increments. U_S_, unique short region; IRL, internal repeat long region; IRS, internal repeat short region; nt, nucleotide.

**FIG 6:**
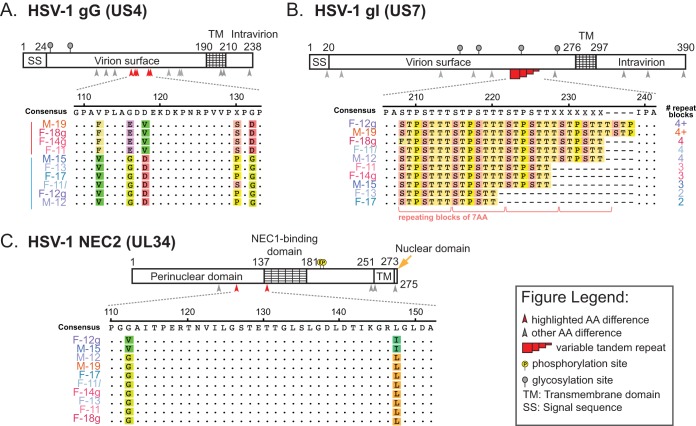
Variations in individual genes provide a better insight into Finnish HSV-1 strain subgroups and of specific cellular phenotypes. Examples are shown here of variation in individual HSV-1 genes that either reflect the Finnish HSV-1 strain subgroups or correlate with and potentially influence specific cellular phenotypes. Diagrams depict three examples of HSV-1 proteins with known functional domains and posttranslational modifications (e.g., phosphorylation and glycosylation). A subset of each protein is highlighted via an amino acid alignment of the 10 clinical HSV-1 isolates. (A) Coding differences in the secreted and virion surface glycoprotein G (gG; US4) correlate with the overall genomic subgroups (clades) shown in Fig. 3. (B) Variation in copy number of a 7-amino-acid (AA) tandem repeat block in glycoprotein I (gI; US7) changes the length of a repeating tract of mucin-type O-linked glycosylation sites, as described previously in clinical isolates of HSV-1 (16, 39). (C) Amino acid differences in the nuclear egress protein NEC2 (UL34) were observed in the two strains (M-15 and F-12g) which showed no detectable extracellular virion release in differentiated neuronal cells (Fig. 1D). See the Discussion for additional insights on the potential phenotypic impacts of the variations shown in panels B and C.

Each viral consensus genome represents the most common nucleotide detected at each position in the viral genome. In contrast, minor variants (MVs) within each viral population are rare alleles, present in <50% of the sequenced reads. These MVs may rise to greater prevalence during viral spread to new niches or hosts or under selective evolutionary pressure. For each viral genome, we examined the number of MVs detected in each genome. Stringent quality control criteria were used to reduce the number of false-positive MVs (see Materials and Methods), and only those MVs detected at ≥2% prevalence were used in this analysis. MVs were found dispersed across each genome (Fig. 5A), mostly in intergenic regions, and were mostly of low frequency (Fig. 5B). A concentration of MVs was found in the internal repeat region, which may result from stochasticity in tandem repeat alignment in this area and/or from the reduced selective pressures on intergenic sequences found in this region. The MVs detected in US7 (gI) occur at the same tandem repeat site as the consensus-level amino acid variations in this gene (Fig. 6). Taken together, these results define the level of interhost (Fig. 4) and intrahost (Fig. 5) variation seen in the Finnish HSV-1 strains described here.

### Patterns of variation in Finnish HSV-1 strains.

Finally, we considered whether any of the observed patterns of genomic or phenotypic variation could be linked to fine-scale coding variations in these 10 Finnish HSV-1 isolates. The genomic subgroups detected by RFLP (Fig. 2) and phylogenetic network analysis (Fig. 3) were reflected in several coding variations that correlated with phylogenetic group. For example, coding differences in the secreted and virion surface glycoprotein G (gG; US4) (Fig. 6A) correlated with the two Finnish phylogenetic subgroups (Fig. 3). Other impacts on coding variation may result from changes in copy number at tandem repeats. Variation in tandem repeat length in glycoprotein I (gI; US7) leads to changes in the length of a repeating tract of mucin-type O-linked glycosylation sites (Fig. 6B), akin to that previously described in clinical isolates of HSV-1 (16, 39). Finally, there are detectable patterns of amino acid variation that correlate with phenotypic differences in viral fitness in specific cell types. Coding differences in the nuclear egress complex protein NEC2 (UL34) (Fig. 6C) were observed in the two strains (F-12g and M-15) that showed no detectable extracellular virion release in differentiated neuronal cells (Fig. 1D). The terminally differentiated and nondividing state of these neuronal cells may constitute a sensitized background to detect impacts on virion egress. Taken together, these examples illustrate the type and degree of variation present in these strains and highlight potential genetic insights into viral phenotype.

## DISCUSSION

In this study, we described for the first time HSV-1 genomes from Finland. Two distinct subgroups or clades were observed in the 10 Finnish clinical isolates, with four strains clustering in one clade and six clustering in another. These groups were detectable using both classical RFLP approaches (Fig. 2) and deep-sequencing methods for whole-genome analyses (Fig. 3). These data appear to correlate with previous findings on the colonization of Finland, with migration from Europe in the south/southwest and from Asia in the northeast (24–26). However, we found that phenotypic differences between these isolates were uncoupled from the overall genomic patterns that grouped them into two geographic clades. While plaque morphology was consistent across isolates (data not shown), the amounts of virus production, extracellular virion release, and acyclovir sensitivity differed between isolates and across cell types (Fig. 1 and Table 1). We detected gene-specific patterns of genetic variation that may impact protein function. Future studies will need to dissect individual genetic variations in each viral isolate in order to test their precise impact(s) on viral fitness.

When analyzing the replication capabilities of these recently isolated clinical samples in multiple cell types, we found that they replicated to lower overall titers and produced fewer extracellular virions than the lab-adapted HSV-1 strain 17+, regardless of cell type (Fig. 1). This difference was more pronounced in nonhuman primate epithelial and human keratinocyte cells and was subtler in neuronal precursors and differentiated neuronal cells. There did not appear to be any clear differences in titers or extracellular virus production levels between geographic clades. There also seemed to be no noticeable differences in plaque morphologies among these samples, with plaque sizes and cellular cytopathic effects being similar across all samples (data not shown). Although we report findings in a number of cell lines in this study, it will be important for future studies to examine each virus and cell type pairing separately since these data demonstrate that maximal viral fitness in one cell type is not generally predictive of fitness in another.

We observed several potential connections between genetic differences and phenotypic differences. For instance, we detected an amino acid difference in the UL34 (NEC2) binding domain that is shared by F-12g and M-15 (Fig. 6C). Previous work has shown that the viral proteins UL31 (NEC1) and UL34 (NEC2) form a nuclear egress complex that localizes to the perinuclear space and plays a critical role in egress of viral capsids from the nucleus (40, 41). In these two clinical isolates (F-12g and M-15), there was no measurable amount of extracellular virus produced in differentiated neuronal cells (Fig. 1D). Further studies will be needed to examine whether this phenotype is linked to a disruption of NEC1 and NEC2 binding and impaired nuclear egress for isolates F-12g and M-15. Variation in the copy number of tandem repeats is yet another way that HSV-1 isolates can generate genetic and potential phenotypic variation (9, 16, 42–44). Glycoprotein I (gI; US7) contains a mucin-like domain with repeating units of serine, threonine, and proline (Fig. 6B). This domain varies in length across these isolates. This domain has been previously shown to serve as a site of O-linked glycosylation, with longer tracts (e.g., 8 repeating blocks of 7 amino acids each) being more heavily glycosylated than short tracts (e.g., 2 repeating blocks) (16, 39). A prior study of over 80 Swedish HSV-1 isolates found roughly equal distributions of isolates with two, three, or four repeating blocks in this mucin-like gI domain (16, 39). Half of the Finnish HSV-1 isolates analyzed here have at least four repeating units, suggesting the potential for gI to be more heavily glycosylated in these isolates. These data generate hypotheses for future investigations of genotype-phenotype links in these and other new clinical isolates.

This study provides one of the first combinations of phenotypic analyses that span multiple cell types, alongside full comparative genomic analyses that span both phylogenetic and gene-specific analyses. One comparison not yet explored in this or prior studies is the specific pairing of each virus with cells derived from the human source of each isolate. This approach is now technically feasible, using induced pluripotent stem cell technology to generate specific cell types from a single source such as a buccal swab. The rich genealogical databases in Finland suggest an opportunity not only to pair these analyses at the human cell and virus level but also to link observed phenotypes of cellular infection to each patient’s clinical, genetic, and/or familial history of herpesvirus disease. Future studies may also seek to link human interaction data, for instance, contact networks and/or travel history, to infer how human movement and interaction patterns influence pathogen spread and population dynamics. We anticipate that in the future this approach would not only yield fruitful insights into direct clinical outcomes of herpesvirus disease but also generate hypotheses about herpesvirus comorbidities.

## MATERIALS AND METHODS

### Virus isolates and virus stock propagation.

Clinical isolates were obtained from anonymous coded diagnostic samples from herpes lesions representing currently circulating viral strains in Finland (Table 1). The virus diagnostic unit of the Turku University Hospital receives viral culture samples from the whole country except for the Helsinki-Uusimaa region (capital Helsinki area and southeastern Finland). Recent immigrations to Finland were not represented in the sample material. Approval for the study of anonymous HSV isolates was provided by the Turku University Central Hospital (permit number J10/17).

An immunoperoxidase rapid culture assay (45) was used to type the viruses as HSV-1, which was confirmed by an HSV type-specific gD (US6) gene-based PCR test (46). Viruses were initially propagated on Vero cells (African green monkey kidney cells; ATCC), maintained in Dulbecco's modified Eagle’s medium (DMEM) with 2% fetal calf serum and gentamicin. A stock was made by addition of 3 ml of 9% autoclaved skimmed milk (Valio, Finland) onto 5 ml of the culture medium and subsequent freezing. The cells and the medium were collected and combined upon thawing and were frozen and thawed for two further rounds before aliquoting. The viral titer was determined by plaque titration on Vero cells as described before (47). Parallel aliquots were used for further viral culture studies and for preparation of viral nucleocapsid DNA.

### Statistical methods.

For viral production data, SPSS Statistics, version 20 (IBM, Armonk NY, USA), software was used to perform statistical analyses. A nonparametric Mann-Whitney U test was used to calculate statistical significances. The threshold for significance was set to *P* value of <0.05.

### Viral genomic DNA isolation.

The viral genomic DNA was prepared from isolated viral nucleocapsids as described previously (7, 48). In brief, viral stock collected from the first or second passage in Vero cells was used to infect ∼1 × 10^8^ HaCaT cells (Department of Dentistry, University of Turku [49]) at a multiplicity of infection (MOI) of 0.1 to 1 PFU/cell, and the infection was allowed to proceed to completion at +35°C (1 to 3 days). The cells were collected, washed with phosphate-buffered saline (PBS), and suspended in LCM buffer (0.125 M KCl, 30 mM Tris, pH 7.4, 5 mM MgCl_2_, 0.5 mM EDTA, 0.5% Nonidet P-40, with 0.6 mM beta-mercaptoethanol). After two successive extractions with Freon (1,1,2-trichloro-1,2,2-trifluoroethane; Sigma-Aldrich), the extracts were added on top of the layers in LCM buffer with 45% and 5% glycerol and ultracentrifuged at 77,000 × *g* for 1 h at +4°C (SW41Ti rotor; Beckman Coulter). Viral nucleocapsids were recovered from the bottom of the ultracentrifuge tube, and the DNA was prepared by treatment with proteinase K and SDS, followed by repeated extractions with phenol-chloroform and ethanol precipitation. The DNA content and purity were observed by spectrophotometry and by agarose gel electrophoresis after restriction enzyme digestions.

### Cell culture.

Vero cells (ATCC, Manassas, VA) were propagated in M199 medium supplemented with 5% fetal bovine serum and gentamicin. HaCaT cells (Department of Dentistry, University of Turku [49]) were propagated in DMEM with HEPES buffer, supplemented with 7% fetal bovine serum and gentamicin. SH-SY5Y neuroblastoma cells (K. Åkerman, Åbo Akademi University, Turku, Finland) were propagated in DMEM (high glucose) supplemented with 10% fetal bovine serum, 2 mM l-glutamine, and gentamicin. Initial differentiation of SH-SY5Y cells involved culture for 10 days in DMEM/F-12 medium containing 5% fetal bovine serum, 10 μM all-*trans* retinoic acid (Sigma), 2 mM l-glutamine, and gentamicin. Thereafter SH-SY5Y cells were transferred on Matrigel-coated (BD) 96-well plates, and the medium was changed to serum-free DMEM/F-12 medium containing 10 μM all-*trans* retinoic acid (Sigma), 0.5 μg/ml of brain-derived neurotrophic factor (Millipore), 2 mM l-glutamine, and gentamicin.

### Acyclovir resistance testing.

The sensitivity of each HSV strain to acyclovir was tested in a microplate format. Vero cells grown on 96-well cell culture plates were treated with cell culture medium (DMEM with 5% fetal bovine serum) supplemented with acyclovir (ACV; Sigma), in concentrations of 128 μg/ml to 0.03125 μg/ml (1:4 serial dilutions). Duplicate wells containing each ACV dilution and wells without ACV were infected with 100 PFU of each virus. As a control, the HSV-1 Δ305 virus was included; it is resistant to ACV due to deletion of its thymidine kinase gene (50). Infected cells were incubated at +37°C in 5% CO_2_ for 72 h before fixation with methanol and staining with crystal violet. The reduction of plaque numbers at each ACV dilution was observed in comparison to levels in wells infected without ACV. A logistic fit curve was used for determination of IC_50_ values. A virus with an IC_50_ value of over 2 μg/ml (of ACV) was considered resistant (51).

### Image acquisition.

Photomicrographic images of viral plaques were obtained using a Zeiss Primovert inverted microscope with Plan-Achromat 4× and 10× objectives, recorded using an AxioCam ERc 5s camera, and analyzed using Zeiss ZEN 2012 software.

### Next-generation sequencing.

Viral nucleocapsid DNA was sheared on a Covaris M220 (parameters: 60-s duration, peak power of 50, 10% duty cycle, 4°C). We used an Illumina TruSeq DNA sample prep kit to prepare barcoded sequencing libraries, according to the manufacturer’s protocol for low-throughput sample handling. Libraries were quantified and assessed by Qubit (Invitrogen, CA), Bioanalyzer (Agilent), and library adapter quantitative PCR (qPCR) (KAPA Biosystems). Illumina MiSeq paired-end sequencing (2 by 300 bp) was completed according to the manufacturer’s recommendations, using a 17-pM library concentration.

A consensus viral genome for each strain was assembled using a *de novo* viral genome assembly (VirGA) workflow (10). This approach begins with quality control, including removal of contaminating host sequences, adapters from library preparation, and imaging artifacts. Next, VirGA iterates through multiple *de novo* assemblies using SSAKE, and these are then combined into longer blocks of sequence (contigs) using Celera and GapFiller. VirGA uses Mugsy alignment to match these contigs to the HSV-1 reference genome (strain 17; GenBank accession JN555585). The best-matched contigs are stitched into a single consensus genome, which is then annotated and subjected to additional quality control measures. These include an examination of coverage (sequence read) depth, detection of minor variants within each consensus genome, and manual inspection of gaps and low-coverage areas. GenBank accession numbers are given below and in Table 2.

### Intrastrain minor-variant detection.

Minor-variant (MV) positions within each *de novo*-assembled genome were determined using VarScan, version 2.2.11, as previously described (19, 52). Conservative variant calling parameters to eliminate sequencing-induced errors were set as follows: minimum allele frequency, ≥0.02; base call quality, ≥20; read depth at the position, ≥100; independent reads supporting minor allele, ≥5. MVs containing ≥90% unidirectional strand support were excluded from further analyses (53, 54), as were those occurring within 10 bp of one another. MVs passing quality control were mapped to respective genomes and assessed for mutational effect using SnpEff and SnpSift (55, 56).

### Phylogenetic and recombination analyses.

DNA sequences were aligned using the Kalign algorithm included in eBioTools. To avoid inferences caused by false phylogenetic signals, all gap and repeat regions were excluded prior to this analysis. Repeat regions not leading to gaps were also excluded since these may contain single nucleotide differences that have been shuffled into new positions by random aspects of tandem repeat alignment. Furthermore, nucleotides that were identified as Ns in GenBank-derived strains were excluded, along with the corresponding aligned nucleotides in remaining sequences. Complete genomes in GenBank harboring long stretches of Ns were not included in the analysis (e.g., strain B3x_1_5). The network is based on an alignment of 116,610 nucleotides, which is more than 75% of the entire HSV-1 genome.

The phylogenetic network was constructed by using SplitsTree4. The network is based on the Neighbor-Net method with ordinary least squares variance depicted as a rooted equal-angle SplitsTree. A recombination test on the complete data set was performed by using the Phi test for recombination implemented in SplitsTree4. Bootstrapping values greater than 80 are shown for clades with three or more strains.

### Restriction fragment length polymorphism (RFLP) analysis.

A cytoplasmic viral DNA preparation was modified from the protocol described by Igarashi et al. (57). Subconfluent Vero cell cultures were infected at an MOI of 0.1 and incubated at +34°C for 2 days until the cytopathic effect was complete. The cells were collected in 150 mM NaCl, 10 mM Tris, pH 7.6, and 1.5 mM MgCl buffer, with 0.1% Nonidet P-40. The nuclei were pelleted, and DNA was extracted from the supernatant by two successive extractions with phenol-chloroform. DNA was recovered by ethanol precipitation. Each viral DNA sample (12.5 µg) was diluted in 15 µl of solution containing sterile H_2_O, FastDigest 10× green buffer, and FastDigest BamHI or SalI restriction enzyme (Thermo Scientific). Electrophoresis was run in 0.8% Tris-borate-EDTA (TBE)-agarose gels, with GeneRuler mix DNA ladder, for 24 h at 45 V before imaging. In order to separate large DNA fragments after SalI digestion, electrophoresis was continued for an additional 36 h at 30 V.

### Data availability.

Data have been deposited in GenBank under accession numbers MH999842 to MH999851.

## Supplementary Material

Supplemental file 1
