# Supplementary material for: Comparison of Herpes Simplex Virus 1 Strains Circulating in Finland Demonstrates the Uncoupling of Whole-Genome Relatedness and Phenotypic Outcomes of Viral Infection
Source: J Virol. 2019 Apr 3;93(8):e01824-18. doi: 10.1128/JVI.01824-18 (PMC6450105; doi:10.1128/JVI.01824-18)
Supplement: Supplemental file 1 [file JVI.01824-18-s0001.pdf]

Supplemental Tables for

Comparison of HSV-1 strains circulating in Finland demonstrates the uncoupling of whole-genome relatedness and phenotypic outcomes of viral infection

Christopher D. Bowen, Henrik Paavilainen, Daniel W. Renner, Jussi Palomäki, Jenni Lehtinen, Tytti Vuorinen, Peter Norberg, Veijo Hukkanen, Moriah L. Szpara

Corresponding Author: Moriah L. Szpara, moriah@psu.edu

Table S1: List of previously published HSV-1 genomes used for phylogenetic analyses

Virus Isolate	Country (with location detail, if available)	GenBank Accession #	References
H1211 / F-11*	Finland	MH999843	(29), present
H1215 / M-15*	Finland	MH999846	(29), present
H12113 / F-13*	Finland	MH999842	present
H12114 / F-14g*	Finland	MH999844	(29), present
H12117 / F-17*	Finland	MH999845	(29), present
H12118 / F-18g*	Finland	MH999847	(29), present
H1311 / F11l*	Finland	MH999848	present
H1312 / M-12*	Finland	MH999849	present
H1412 / F-12g*	Finland	MH999851	present
H15119 / M-19*	Finland	MH999850	present
SC16	Spain (Madrid)	KX946970	(34)
172/2010	Germany	LT594105	(12)
2158/2007	Germany	LT594106	(12)
3083/2008	Germany	LT594107	(12)
1319/2005	Germany	LT594108	(12)
270/2007	Germany	LT594109	(12)
66/2007	Germany	LT594110	(12)
1394/2005	Germany	LT594111	(12)
369/2007	Germany	LT594112	(12)
160/1982	Germany	LT594192	(12)
132/1998	Germany	LT594457	(12)
L2	Russia (Moscow)	KT780616	(35)
B^3x1.1	U.S.A. (Bronx, NY)	KU310657	(36)
B^3x1.2	U.S.A. (Bronx, NY)	KU310658	(36)
B^3x1.3	U.S.A. (Bronx, NY)	KU310659	(36)
B^3x1.4	U.S.A. (Bronx, NY)	KU310660	(36)
B^3x1.5	U.S.A. (Bronx, NY)	KU310661	(36)
H193	U.S.A.	KT425108	n/a
KOS79	U.S.A. (Madison, WI)	KT425109	(11)
CJ994	U.S.A. (Madison, WI)	KR011283	(20)
HSV-1/0116209/India/2011	India	KJ847330	(37)
H166	U.S.A.	KM222726	(10)
H166syn	U.S.A.	KM222727	(10)

Virus Isolate	Country (with location detail, if available)	GenBank Accession #	References
RE	U.S.A.	KF498959	n/a
OD4	U.S.A. (Madison, WI)	JN420342	(8)
17	U.K. (Glasgow)	JN555585	(9)
CR38	China (Shenyang)	HM585508	(9)
E03	Kenya (Nairobi)	HM585509	(9)
E06	Kenya (Nairobi)	HM585496	(9)
E07	Kenya (Nairobi)	HM585497	(9)
E08	Kenya (Nairobi)	HM585498	(9)
E10	Kenya (Nairobi)	HM585499	(9)
E11	Kenya (Nairobi)	HM585500	(9)
E12	Kenya (Nairobi)	HM585501	(9)
E13	Kenya (Nairobi)	HM585502	(9)
E14	Kenya (Nairobi)	HM585510	(9)
E15	Kenya (Nairobi)	HM585503	(9)
E19	Kenya (Nairobi)	HM585511	(9)
E22	Kenya (Nairobi)	HM585504	(9)
E23	Kenya (Nairobi)	HM585505	(9)
E25	Kenya (Nairobi)	HM585506	(9)
E35	Kenya (Nairobi)	HM585507	(9)
R11	South Korea (Seoul)	HM585514	(9)
R62	South Korea (Seoul)	HM585515	(9)
S23	Japan (Sapporo)	HM585512	(9)
S25	Japan (Sapporo)	HM585513	(9)
F	U.S.A. (Chicago, IL)	GU734771	(9)
H129	U.S.A. (San Francisco, CA)	GU734772	(9)
McKrae	U.S.A. (Gainesville, FL)	JQ730035, JX142173	(9)
KOS	U.S.A. (Houston, TX)	JQ673480, JQ780693	(9)
HF10	U.S.A. (New York, NY)	DQ889502	(9)
Ty_25	Japan	MH999840	n/a
Ty_148	Japan	MH999841	n/a
K_86	Japan	MH999839	n/a
K_47	Japan	MH999838	n/a
7-hse[†]	Sweden or U.S.A.	SRX056767	(38)
7862[†]	Sweden or U.S.A.	SRX056770	(38)
3355[†]	Sweden or U.S.A.	SRX056769	(38)
2762[†]	Sweden or U.S.A.	SRX056768	(38)
90237[†]	Sweden or U.S.A.	SRX056772	(38)
E4[†]	Sweden or U.S.A.	SRX056773	(38)
78326[†]	Sweden or U.S.A.	SRX056771	(38)
4-J1037[†]	Sweden or U.S.A.	SRX056760	(38)
J1061[†]	Sweden or U.S.A.	SRX056774	(38)
5-J1061	Sweden or U.S.A.	n/a	(38)

[*] newly sequenced strains from Finland in this study.

[†] Sequence data deposited in GenBank short read archive

References (Reference numbers match that used in table and main manuscript)

8. Kolb AW, Adams M, Cabot EL, Craven M, Brandt CR. 2011. Multiplex sequencing of seven ocular herpes simplex virus type-1 genomes: phy- logeny, sequence variability, and SNP distribution. Invest Ophthalmol Vis Sci 52:9061–9073. https://doi.org/10.1167/iovs.11-7812.

9. Szpara ML, Gatherer D, Ochoa A, Greenbaum B, Dolan A, Bowden RJ, Enquist LW, Legendre M, Davison AJ. 2014. Evolution and diversity in human herpes simplex virus genomes. J Virol 88:1209 –1227. https://doi.org/10.1128/JVI.01987-13.

10. Parsons LR, Tafuri YR, Shreve JT, Bowen CD, Shipley MM, Enquist LW, Szpara ML. 2015. Rapid genome assembly and comparison decode intrastrain variation in human alphaherpesviruses. mBio 6:e02213-14. https://doi.org/10.1128/mBio.02213-14.

11. Bowen CD, Renner DW, Shreve JT, Tafuri Y, Payne KM, Dix RD, Kinchington PR, Gatherer D, Szpara ML. 2016. Viral forensic genomics reveals the relat- edness of classic herpes simplex virus strains KOS, KOS63, and KOS79. Virology 492:179 –186. https://doi.org/10.1016/j.virol.2016.02.013.

12. Pfaff F, Groth M, Sauerbrei A, Zell R. 2016. Genotyping of herpes simplex virus type 1 by whole genome sequencing. J Gen Virol 97:2732–2741. https://doi.org/10.1099/jgv.0.000589.

20. Lee K, Kolb AW, Sverchkov Y, Cuellar JA, Craven M, Brandt CR. 2015. Recombination analysis of herpes simplex virus 1 reveals a bias toward GC content and the inverted repeat regions. J Virol 89:7214 –7223. https://doi.org/10.1128/JVI.00880-15.

29. Paavilainen H, Lehtinen J, Romanovskaya A, Nygårdas M, Bamford DH, Poranen MM, Hukkanen V. 2016. Inhibition of clinical pathogenic herpes simplex virus 1 strains with enzymatically created siRNA pools. J Med Virol 88:2196 –2205. https://doi.org/10.1002/jmv.24578.

34. Rastrojo A, López-Muñoz AD, Alcamí A. 2017. Genome sequence of herpes simplex virus 1 strain SC16. Genome Announc 5:e01392-16. https://doi.org/10.1128/genomeA.01392-16.

35. Skoblov MY, Lavrov AV, Bragin AG, Zubtsov DA, Andronova VL, Galegov GA, Skoblov YS. 2017. The genome nucleotide sequence of herpes simplex virus 1 strain L2. Russ J Bioorg Chem 43:140 –142. https://doi.org/10.1134/S1068162016060133.

36. Petro CD, Weinrick B, Khajoueinejad N, Burn C, Sellers R, Jacobs WR, Herold BC. 2016. HSV-2 ΔgD elicits FcyR-effector antibodies that protect against clinical isolates. JCI Insight 1:e88529. https://doi.org/10.1172/jci.insight.88529.

37. Bondre VP, Sankararaman V, Andhare V, Tupekar M, Sapkal GN. 2016. Genetic characterization of human herpesvirus type 1: Full-length genome sequence of strain obtained from an encephalitis case from India. Indian J Med Res 144:750 –760. https://doi.org/10.4103/ijmr.IJMR_747_14.

38. Norberg P, Tyler S, Severini A, Whitley R, Liljeqvist J-A, Bergstrom T. 2011. A genome-wide comparative evolutionary analysis of herpes simplex virus type 1 and varicella zoster virus. PLoS One 6:1– 8. https://doi.org/10.1371/journal.pone.0022527.

Table S2: Number of nucleotide and amino acid (AA) differences observed in each set of Finnish or worldwide published HSV1 genomes.

Gene*	ORF length in NT^	protein length (#AA)	10 Finnish # NT diff.^	10 Finnish # AA changes	10 Finnish %AA diff.	10 Finnish dN/dS	All HSV1 # NT diff.	All HSV1 # AA changes	All HSV1 %AA diff.	All HSV1 dN/dS ratio
UL1	675	224	22	12	5.4	1.20	58	31	13.8	1.15
UL2	1005	334	24	8	2.4	0.50	62	28	8.4	0.82
UL3	675	224	13	5	2.2	0.63	43	19	8.5	0.79
UL4	600	199	11	2	1.0	0.22	43	18	9.0	0.72
UL5	2649	882	35	13	1.5	0.59	106	38	4.3	0.56
UL6	2031	676	43	13	1.9	0.43	107	30	4.4	0.39
UL7	891	296	24	6	2.0	0.33	47	17	5.7	0.57
UL8	2253	750	44	18	2.4	0.69	124	62	8.3	1.00
UL9	2556	851	21	5	0.6	0.31	110	32	3.8	0.41
UL10	1422	473	25	5	1.1	0.25	81	30	6.3	0.59
UL11	291	96	13	5	5.2	0.63	26	17	17.7	1.89
UL12	1881	626	36	12	1.9	0.50	87	30	4.8	0.53
UL13	1557	518	27	10	1.9	0.59	72	33	6.4	0.85
UL14	660	219	10	6	2.7	1.50	27	15	6.8	1.25
UL15	2208	735	22	3	0.4	0.16	80	16	2.2	0.25
UL16	1122	373	13	3	0.8	0.30	55	19	5.1	0.53
UL17	2112	703	30	12	1.7	0.67	108	39	5.5	0.57
UL18	957	318	12	6	1.9	1.00	41	15	4.7	0.58
UL19	4125	1374	39	7	0.5	0.22	139	40	2.9	0.40
UL20	669	222	7	0	0.0	0.00	29	9	4.1	0.45
UL21	1608	535	20	9	1.7	0.82	70	25	4.7	0.56
UL22	2517	838	21	8	1.0	0.62	120	50	6.0	0.71
UL23	1131	376	21	8	2.1	0.62	63	26	6.9	0.70
UL24	810	269	18	7	2.6	0.64	60	28	10.4	0.88

Gene*	ORF length in NT^	protein length (#AA)	10 Finnish # NT diff.^	10 Finnish # AA changes	10 Finnish %AA diff.	10 Finnish dN/dS	All HSV1 # NT diff.	All HSV1 # AA changes	All HSV1 %AA diff.	All HSV1 dN/dS ratio
UL25	1743	580	19	4	0.7	0.27	74	25	4.3	0.51
UL26	1908	635	40	14	2.2	0.54	92	34	5.4	0.59
UL26.5	990	329	21	8	2.4	0.62	45	17	5.2	0.61
UL27	2715	904	51	14	1.5	0.38	123	40	4.4	0.48
UL28	2358	785	26	6	0.8	0.30	87	23	2.9	0.36
UL29	3591	1196	54	10	0.8	0.23	154	33	2.8	0.27
UL30	3708	1235	32	13	1.1	0.68	154	49	4.0	0.47
UL31	921	306	15	5	1.6	0.50	34	13	4.2	0.62
UL32	1791	596	20	2	0.3	0.11	85	28	4.7	0.49
UL33	393	130	2	1	0.8	1.00	14	4	3.1	0.40
UL34	828	275	15	6	2.2	0.67	37	14	5.1	0.61
UL35	339	112	1	0	0.0	0.00	8	1	0.9	0.14
UL36	9420	3139	166	66	2.1	0.66	1271	440	14.0	0.53
UL37	3372	1123	41	13	1.2	0.46	140	60	5.3	0.75
UL38	1398	465	18	8	1.7	0.80	70	30	6.5	0.75
UL39	3414	1137	42	13	1.1	0.45	157	61	5.4	0.64
UL40	1023	340	10	3	0.9	0.43	38	12	3.5	0.46
UL41	1470	489	16	3	0.6	0.23	52	22	4.5	0.73
UL42	1467	488	19	10	2.0	1.11	76	39	8.0	1.05
UL43	1254	417	35	17	4.1	0.94	90	50	12.0	1.25
UL44	1536	511	26	16	3.1	1.60	97	55	10.8	1.31
UL45	519	172	4	3	1.7	3.00	22	8	4.7	0.57
UL46	2157	718	33	15	2.1	0.83	111	65	9.1	1.41
UL47	2082	693	29	6	0.9	0.26	81	29	4.2	0.56
UL48	1473	490	18	4	0.8	0.29	69	25	5.1	0.57
UL49	276	301	13	5	1.7	0.63	42	18	6.0	0.75
UL49A	906	91	2	0	0.0	0.00	8	3	3.3	0.60
UL50	1116	371	23	9	2.4	0.64	65	26	7.0	0.67

Gene*	ORF length in NT^	protein length (#AA)	10 Finnish # NT diff.^	10 Finnish # AA changes	10 Finnish %AA diff.	10 Finnish dN/dS	All HSV1 # NT diff.	All HSV1 # AA changes	All HSV1 %AA diff.	All HSV1 dN/dS ratio
UL51	735	244	12	3	1.2	0.33	33	13	5.3	0.65
UL52	3177	1058	43	9	0.9	0.26	129	41	3.9	0.47
UL53	1017	338	16	4	1.2	0.33	36	12	3.6	0.50
UL54	1539	512	11	4	0.8	0.57	73	34	6.6	0.87
UL55	561	186	5	0	0.0	0.00	25	9	4.8	0.56
UL56	705	234	8	4	1.7	1.00	34	19	8.1	1.27
US1	1263	420	22	8	1.9	0.57	75	39	9.3	1.08
US2	876	291	24	6	2.1	0.33	44	14	4.8	0.47
US3	1446	481	14	5	1.0	0.56	57	21	4.4	0.58
US4	717	238	26	13	5.5	1.00	68	36	15.1	1.13
US5	279	92	10	7	7.6	2.33	25	17	18.5	2.13
US6	1185	394	18	4	1.0	0.29	67	18	4.6	0.37
US7	1173	390	28	12	3.1	0.75	119	57	14.6	0.92
US8	1653	550	30	13	2.4	0.76	79	38	23.9	0.93
US8A	480	159	3	3	1.9	1.00	49	15	2.7	0.44
US9	273	90	1	1	1.1	1.00	12	4	4.4	0.50
US10	939	312	11	5	1.6	0.83	65	29	9.3	0.81
US11	486	161	1	1	0.6	1.00	42	19	11.8	0.83
US12	267	88	4	2	2.3	1.00	17	10	11.4	1.43
RL1	747	248	25	11	4.4	0.79	422	90	36.3	0.27
RL2	2328	775	44	20	2.6	0.83	180	91	11.7	1.02
RS1	3897	1298	62	30	2.3	0.94	345	207	15.9	1.50

* See **Table S1** for list of strain names and genome accessions for comparisons of 10 Finnish and all published HSV1 genomes. See **Table S3** for list of strains excluded from selected gene alignments due to missing data in GenBank.

^ Abbreviations: ORF, open reading frame; NT, nucleotide; AA, amino acid; diff., difference; %, percent; dN/dS, ratio of nonsynonymous to synonymous nucleotide changes

Table S3: HSV-1 strains used to calculate nucleotide and amino acid (AA) differences in Table S2 and Figure 4.

Gene	# of strains in nucleotide alignment	# of strains in AA alignment	Strains not used in nucleotide and AA alignments*
UL7	60	60	B^3x1.1, B^3x1.2, B^3x1.3, B^3x1.4, B^3x1.5
UL12	61	61	B^3x1.3, B^3x1.4, B^3x1.5, RE
UL13	58	58	E06, E25, E11, OD4, B^3x1.3, B^3x1.4, B^3x1.5
UL15	60	60	B^3x1.1, B^3x1.2, B^3x1.3, B^3x1.4, B^3x1.5
UL19	61	61	B^3x1.3, B^3x1.4, B^3x1.5, RE
UL26.5	61	61	B^3x1.3, B^3x1.4, B^3x1.5, HF10
UL41	61	61	B^3x1.3, B^3x1.4, B^3x1.5, OD4
UL43	61	61	B^3x1.3, B^3x1.4, B^3x1.5, HF10
UL48	60	60	B^3x1.1, B^3x1.2, B^3x1.3, B^3x1.4, B^3x1.5
UL49A	51	51	B^3x1.3, B^3x1.4, B^3x1.5, HF10, 66_2007, 369_2007, 160_1982, 1394_2005, 132_1998, 3083_2008, 2158_2007, 172_2010, 270_2007, 1319_2005
UL55	59	59	E35, E13, KUTy25, B^3x1.3, B^3x1.4, B^3x1.5
UL56	60	60	B^3x1.3, B^3x1.4, B^3x1.5, E35, HF10
US1	60	60	B^3x1.1, B^3x1.2, B^3x1.3, B^3x1.4, B^3x1.5
US8A	61	61	HF10, B^3x1.3, B^3x1.4, B^3x1.5
RL1	58	58	B^3x1.3, B^3x1.4, B^3x1.5, OD4, 1394_2005, CJ994, L2
RL2	61	61	B^3x1.3, B^3x1.4, B^3x1.5, B^3x1.2
RS1	59	59	B^3x1.3, B^3x1.4, B^3x1.5, S23, CJ994, L2
All other genes	62	62	B^3x1.3, B^3x1.4, B^3x1.5

* Strains were excluded due to missing data (e.g. due to sequencing gaps) or annotations in GenBank.
